# Expression profile of inflammasome genes in individuals with Down syndrome

**DOI:** 10.1590/1678-4685-GMB-2023-0339

**Published:** 2024-09-09

**Authors:** Juliana Vieira de Barros Arcoverde, Carla Fernandes dos Santos, Maria Cecília Magalhães Luckwu, Raysa Samanta Moraes Laranjeira, Aldianne Milene dos Santos Barbosa, Thays Maria Costa de Lucena, Jaqueline de Azevêdo Silva, Neide Santos

**Affiliations:** 1 Universidade Federal de Pernambuco, Departamento de Genética, Recife, PE, Brazil.; 2 Universidade Federal de Pernambuco, Instituto Keizo Asami (iLIKA), Recife, PE, Brazil.

**Keywords:** Down syndrome, inflammasome, expression

## Abstract

Down syndrome (DS), affecting 1 in 700 live births, is the most prevalent chromosomal disorder among newborns. Recognizable by classical clinical features, patients with DS are susceptible to various immunological misbalances. Inflammasome is (mis)activated in several immune-mediated diseases, however studies on individuals with DS are lacking. The present study evaluated the gene expression of *NLRP1, NLRP3* and *IL-1*β in individuals with DS, aiming to understand their susceptibility to immune-mediated diseases. In addition, we assessed whether the individuals with DS present a differential inflammatory response after *in vitro* infection using PBMCs. For the gene expression assay, 20 individuals with DS and 15 healthy individuals for the control group (CT) were included, while the *in vitro* infection assay included 10 subjects. mRNA levels from individuals with DS group showed 1.9-fold change (FC) downregulation for *NLRP1* (p=0.0001), but no differences for *NLRP3* and *IL1*β. We did not observe significant differences between lipopolysaccharide (LPS)-treated and untreated cells in our *in vitro* assays. The differential expression of *NLRP1* in individuals with DS suggests a potential association with susceptibility to the development of immune-mediated diseases, but further analysis is needed to confirm this relationship.

## Introduction

Down syndrome (DS) or trisomy 21 (OMIM #190685, Online Mendelian Inheritance in Man, an Online Catalog of Human Genes and Genetic Disorders) is a genetic condition resulting from the presence and expression of three copies of genes located on chromosome 21. It is the most common viable chromosomal aneuploidy in humans (Migliore et al., 2009; Dieudonne et al., 2020; Santos et al., 2021). In Brazil, DS occurs in approximately 1/700 newborns, and affects individuals of all races and ethnic groups (Yaqoob et al., 2019; Bermudez et al., 2021).

Trisomy 21 is primarily attributed to meiotic non-disjunction, resulting in a karyotype with the presence of 47 chromosomes (a condition known as free trisomy) in approximately 95% of the cases. Other alterations include chromosomal mosaicism (2 to 4% of cases), Robertsonian translocation involving chromosome 21 and another acrocentric chromosome (occurring in between 1 and 3% of cases), and chromosomal rearrangements involving the long arm of chromosome 21 (denoted as the critical region of DS and called partial trisomy) (Oster-Granite et al., 2011; Coppedè, 2016; Bull, 2020). Cytogenetic analysis has an important role not only in confirming the diagnosis of DS, but also in predicting the risk of recurrence and providing essential information for future genetic counseling (Narayanan et al., 2013).

Immune-mediated diseases in individuals with DS often lead to complications that can reduce life expectancy, including increasing susceptibility to infections, hematological malignancies and autoimmunity conditions (Ferrari and Stagi, 2021). DS is well recognized for its association with multiple immunological alterations. However, diagnosing autoimmune diseases in individuals with DS can be complicated due to the masking effects of the syndrome’s underlying characteristics, such as failure of growth, short stature and delayed puberty (Christiakov, 2007; Bull, 2020; Jones et al., 2020).

In the immunologic context of DS, interferons (IFN-Is), cytokines with inflammatory and antiviral functions, stand out. Chromosome 21 encodes four subunits of *IFN* receptors (IFN-Rs): *IFNAR1*, *IFNAR2*, *IFNGR2* and *IL10RB*. The 1.5-fold increase in copy number of *IFNAR1* and *IFNAR2* found in individuals with DS has been shown to be an important factor in neurodegenerative, endocrine, renal and gastrointestinal disorders with common autoimmune etiologies of the syndrome (Jagadeesh et al., 2020; Malle and Bogunovic, 2021).

The knowledge in inflammasome pathway and its components has demonstrated its important roles in various diseases, both autoinflammatory and autoimmune conditions (Kahlenberg and Kang, 2021). However, there is a knowledge gap regarding the inflammasome’s role in individuals with DS, a population known for their heightened susceptibility to immune-mediated diseases, which makes our study a pioneer on the subject.

Inflammasomes play an important and vital role in the activation and subsequent release of pro-inflammatory cytokines, exhibiting high expression in immune cells (Guo et al., 2016; Bortolotti et al., 2018; Blevins et al., 2022). These molecular complexes are responsible for regulating the immune response to both exogenous (Pathogen-Associated Molecular Patterns, PAMPs) and endogenous (Damage-Associated Molecular Patterns, DAMPs factors. Dysregulation of inflammasome activation has been associated with the development of various conditions, including cancer, autoimmune diseases, metabolic disorders and neurodegenerative diseases (Broz and Dixit, 2016; Sönmez and Özen, 2017; Bortolotti *et al*., 2018). Recent insights from Alves-Hanna et al. (2023) have shed light on the potential mechanisms by which inflammasome dysregulation can contribute to leukemogenesis. Additionally, they described potential therapeutic targets directed at inflammasomes in the context of leukemia. In patients with DS, who exhibit a significantly higher incidence of acute leukemia, particularly acute megakaryoblastic leukemia (AMKL) and acute lymphoblastic leukemia (ALL), the role of inflammasomes appears to be particularly pertinent.

Individuals with DS have an increased risk of developing inflammatory, infectious, and autoimmune diseases, which can significantly influence their quality of life and expectancy. However, the precise mechanism contributing to the development of these clinical conditions remains not yet well understood. Therefore, the objective of our study was to analyze and compare the expression profile of the inflammasome genes *NLRP1* (NLR, pyrin domain containing 1), *NLRP3* (NLR, pyrin domain containing 3) and *IL-1β* (Interleukin 1 β) in individuals with DS and in healthy controls, as well as analyzing the expression profile of these genes in peripheral blood mononuclear cells (PBMCs) stimulated and not stimulated with lipopolysaccharide (LPS) in these individuals, aiming to understand their susceptibility to immune-mediated diseases and whether they present differential inflammatory response after *in vitro* infection.

## Subjects and Methods

### Patients and controls

The individuals with DS were recruited from the University Group for Child Rehabilitation (Grupo Universitário de Reabilitação Infantil, GURI) and a group formed by parents and guardians of individuals with DS. Our study comprised a sample of 68 individuals with DS. From those, 20 individuals agreed to voluntarily participate in our research and were included in the gene expression assay, with a mean age of 10.9 years (ages ranged from 8 to 14 years ± 1.6). For the cell culture assay, we recruited 10 individuals with DS to use *ex vivo* cells (peripheral mononuclear cells - PBMC) and assess before and after stimulation with lipopolysaccharide (LPS). 

The healthy control group (CT) consisted of 15 children and adolescents, with a mean age of 11.7 years (ranging from 8 to 14 years ± 2.7). These participants did not have DS nor prior history of autoimmune or chronic inflammatory diseases and grew out of a spontaneous demand for volunteers. All individuals in the study come from the state of Pernambuco (PE), Brazil.

Our study was approved by the Research Ethics Committee of the Health Sciences Center of the Federal University of Pernambuco (nº 32478820.0.0000.5208). All legal guardians of the individuals who participated in this research provided their informed consent by signing the Informed Consent Form. All participants voluntarily contributed to the research.

### Lymphocyte culture and G-banding

Cytological preparations for chromosomal analyzes were obtained from lymphocyte cultures, around 20 metaphases were analyzed with G-banding for each individual and in the case of chromosomal mosaicism, around 30 metaphases were analyzed per individual. The chromosomes were identified and classified according to the International System for Human Cytogenetic Nomenclature (ISCN) of 2020. The collection of biological material from individuals with DS and the CT occurred between August 2021 to July 2023.

### Cell culture, RNA extraction and cDNA synthesis

For the cell culture, we isolated peripheral blood mononuclear cells (PBMCs) from 15 mL of venous blood collected from 10 individuals with DS, using tubes containing heparin as an anticoagulant. PBMCs were isolated from whole blood using Histopaque - 1077 (Sigma). A total of 5 × 10^6^ PBMCs per well were cultured in RPMI 1640 (Gibco) supplemented with 10% fetal bovine serum (Invitrogen, Life Technology, USA). The PBMCs were cultivated in 12-well plates within an incubator with 5% CO_2_ at 37 °C. The culture for each individual was divided into two experimental stages: (1) Negative culture - without stimulation (lasting 18 hours); (2) LPS stimulation (4 hours). Inflammation was induced by exposing the cells to LPS at a concentration of 50 ng/ml (Sigma).

Total RNA was isolated from PBMCs and peripheral blood using the Trizol® reagent according to the manufacturer’s instructions. Subsequently, RNA quantification was carried out using the NanoDropTM equipment, using spectrophotometry, to evaluate both the total concentration and purity of the RNA. The isolated RNA samples were stored in a -80 °C freezer for preservation. 

The synthesis of complementary DNA (cDNA), utilizing a standard RNA concentration of 500 ng, was carried out with the High Capacity cDNA Reverse Transcription Kit (ThermoFisher).

### Gene expression assay

The expression assay was performed using Taqman® type fluorogenic probes for the target genes *NLRP1* (Hs00248187_ m1), *NLRP3* (Hs00918082_m1) and *IL-1β* (Hs01555410_m1). The relative expression of the target genes was normalized by employing the endogenous genes *EF1A* (F- GAGGCTGCT GAGATGGGAAA and R- CGTTCACGCTCAGCTTTCAG) and *HPRT1* (F- ACAGGACTGAACGTCTTGCT and R- GAGCACACAGAGGGCTACAA). These endogenous genes were used using the Sybr Green methodology, selected based on their expression stability according to sample and condition tested and determined based on Genorm. Relative expression analysis was performed using the normalization factor.

### Clinical laboratory evaluations

All individuals who participated in the gene expression assay underwent biochemical examinations in a specialized laboratory. At the time of sample blood collection, their caregivers reported that no medications, supplements, or vaccines had been used in the previous 72 hours. These examinations encompassed the assessment of various parameters, including: Ultrasensitive Thyroid Stimulating Hormone (TSH) and free T4 (Free Thyroxine) levels, to detect thyroid disorders; Glycated Hemoglobin (HbA1c) and Estimated Average Glucose (EMG) measurements - to provide insights into diabetes; and Ultrasensitive C-Reactive Protein (us-CRP) detection, a known acute inflammation marker.

Regarding us-CRP levels, the reference values for risk categorization were as follows: Low risk: <0.1mg/dL; Medium risk: 0.1 to 0.2mg/dL; High risk: >0.2mg/dL; Very high risk: >1mg/dL and Acute phase inflammatory diseases: >=1mg/dL (Myers et al., 2009). The individuals showed no signs or symptoms of infection at the time of sample collection for the study.

Thyroid disease was defined and classified based on the following criteria: subclinical hypothyroidism (SH), for cases where individuals exhibited “high” TSH levels while maintaining normal free T4 levels at the time of diagnosis or without medication, and Overt Hypothyroidism (OH), for cases displaying “high” TSH levels coupled with “low” free T4 levels at any point in time. The categorization of “high” and “low” was determined based on individual reference ranges, with the following reference values: TSH range of 0.30 to 4.20 µIU/mL, and free T4 levels between 0.82 to 1.62 ng/dL. 

The diagnosis of diabetes mellitus was defined when both the laboratory reference values for glycated hemoglobin and estimated mean glucose equaled or exceeded 6.5% and 126 mg/dL, respectively.

### Statistical analysis

Gene expression assays were performed in technical duplicate and the results were presented as group means and standard deviations. Statistical analysis and graphics were carried out using GraphPad software, version 8.0.0. To verify the distribution of the samples, the Shapiro-Wilk normality test was performed. 

In the normalized analyses, parametric tests such as Student’s t-test or One-Way ANOVA were applied, whereas non-parametric tests, the Mann-Whitney or Kruskal-Wallis test, were applied in the non-normalized data. A significance level of p< 0.05 was adopted, with a 95% confidence interval.

The software G*Power version 3.1.9.7 was used to assess the statistical power of the study.

## Results

The cytogenetic analysis of the 68 individuals, comprising 38 men and 30 women, revealed that a total of 60 patients exhibited free trisomy, constituting 88.2% of all cases. Additionally, six patients showed chromosomal mosaicism, accounting for 8.8% of the cases, while two patients were identified with Robertsonian translocation, representing 2.9% of the cases (Table 1). All individuals recruited for the gene expression assays underwent anamnesis and had no known prior conditions of autoimmune, inflammatory or endocrine diseases, as well as diabetes and heart disease.

**Table 1 -:** Cytogenetic data of DS individuals.

Karyotypes	N (%)	Karyotypic group (%)
47,XY,+21	32 (47.1)	88.2%
47,XX,+21	28 (41.2)	
47,XY,+21/46,XY	3 (4.4)	8.8%
47,XX,+21/46,XX	1 (1.5)	
47,XY,+21/47,XXY	1 (1.5)	
47,XX,+mar[13]/47,XX,+21[9]/48,XX,+2mar[3]/49,XX,+3mar[2]/46,XX[3]	1 (1.5)	
46,XY,+21,der(21;22)(q10;q10)	1 (1.5)	2.9%
46,XY,+21,der(14;21)(q10;q10)	1 (1.5)	
Total	68 (100%)	100%

Of the 68 individuals with DS, 20 were also evaluated for serum levels of ultra-sensitive thyroid-stimulating hormone (TSH), free thyroxine (free T4), glycated hemoglobin (HbA1c) and ultra-sensitive protein C (us-CPR) (Table 2). All individuals had a karyotype with free trisomy. According to our findings we identified nine individuals displaying changes in TSH, eight of them compatible with subclinical hypothyroidism and one with overt hypothyroidism; and eight individuals showed changes in us-CPR. Furthermore, one individual had diabetes mellitus. HbA1c and EMG values are available in Table 2 (individual 8).

**Table 2 -:** Serum levels of individuals with DS.

Individual	us-CRP (mg/dL)	TSH (µUI/mL)	Free T4 (ng/dL)	HbA1c (%)	EMG (mg/dL)
**1**	0.05	6.46*	1.04	5.2%	103
**2**	0.02	2.95	1.11	5.3%	104
**3**	<0.02	4.14	1.23	5.5%	110
**4**	0.08	2.1	1.59	5.4%	109
**5**	0.19	1.45	0.92	5.1%	99
**6**	0.02	5.31*	0.86	4.5%	82
**7**	0.63*	5.38*	1.32	5.2%	102
**8**	0.79*	6.73*	1.44	8.0%	183*
**9**	0.41*	3.01	0.94	4.9%	95
**10**	0.17	49.32*	0.77*	5.0%	97
**11**	0.33*	3.11	1.16	5.3%	104
**12**	0.02	4	1.12	5.0%	97
**13**	0.95*	3.61	1.56	5.0%	96
**14**	0.55*	4.47*	1.13	5.6%	114
**15**	0.27*	2.97	1.14	5.8%	119
**16**	0.15	16.96*	1.04	4.6%	86
**17**	0.15	2.76	1.37	4.6%	86
**18**	0.19	11.65*	1.44	5.6%	115
**19**	0.11	4.18	1.21	5.2%	102
**20**	0.29*	9.35*	0.97	5.6%	115

*in us-CRP indicates that the levels corresponded to high risk for acute phase inflammatory diseases, and in the TSH, free T4 and EMG column that the value was outside the range of reference.

**Figure 1-:**
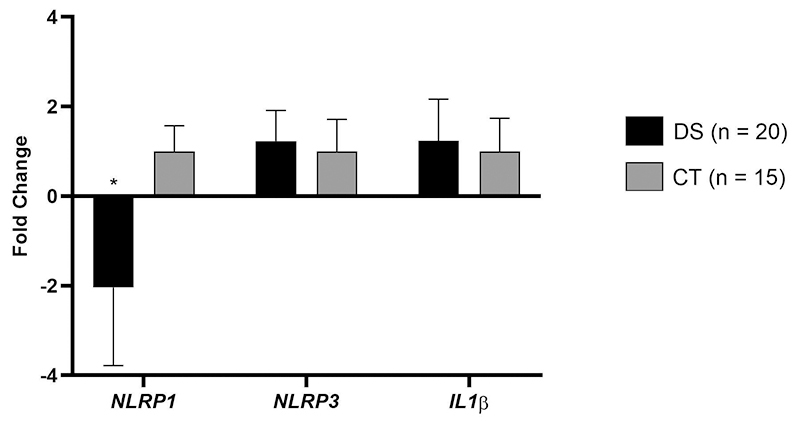
Expression profile of the *NLRP1* (p<0.001), *NLRP3* (p=0.5887) and *IL1β* (p=0.2248) genes in individuals with DS *versus* the healthy control group (CT).

Regarding gene expression assay with peripheral blood, among the 68 individuals with DS, expression from *NLRP1, NLRP3* and *IL-1β* genes were conducted on a subset of 20 individuals (Figure 1). When compared to individuals in the healthy control group (CT, n=15), we observed a downregulation of the *NLRP1* gene of 1.8 times (p<0.0001). In contrast, the *NLRP3* (FC: 1.0; p=0.5887) and *IL1β* (FC: 1.0; p=0.2248) genes showed no differential expression.

**Figure 2 -:**
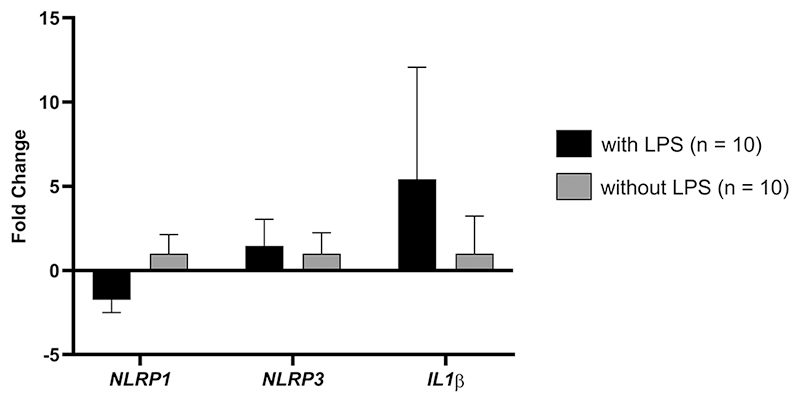
Expression profile of *NLRP1* (p=0.1879)*, NLRP3* (p=0.5889) and *IL1β* (p=0.0075) genes in PBMCs from individuals with DS with and without LPS stimulation.

In the cell culture assay, using PBMCs, mRNA levels for the *NLRP1, NLRP3* and *IL-1β* genes (Figure 2) among individuals with DS (n=10), were downregulated for *NLRP1* +1.7 FC (p=0.1879), and an upregulation for *NLRP3* and *IL-1β* genes of +1.4 FC (p=0.5889) and +5.4 FC (p=0.0075), respectively, when comparing cells treated with LPS stimulation in relation to cells that did not receive the stimulus.

**Figure 3 -:**
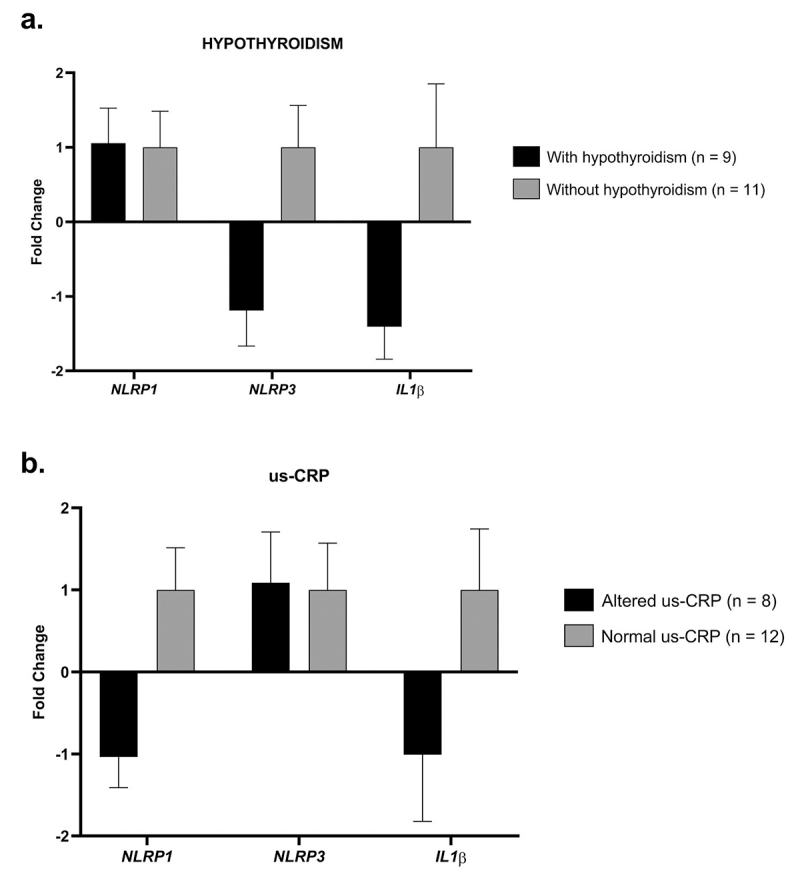
Expression profile of the *NLRP1, NLRP3* and *IL1β* genes. a) Individuals with DS with hypothyroidism versus without hypothyroidism: *NLRP1* (p=0.7912)*, NLRP3 (p=*0.4785*)* and *IL1β* (p=0.3005); b) Individuals with altered us-CRP versus individuals without changes in us-CRP: *NLRP1* (p=0.8587)*, NLRP3* (p=0.7384) and *IL1β* (p=0.9795).

Individuals were stratified into two groups based on changes in TSH and us-CRP. Individuals with hypothyroidism (n=9) (Figure 3 a ), the mRNA levels showed an upregulation for *NLRP1* gene of +1.0 FC (p=0.7912), and a downregulation of the *NLRP3* and *IL-1β* genes of +1.1 FC (p=0.4785) and +1.4 FC (p=0.3005), respectively, when compared with individuals without hypothyroidism (n=11).

For the group with changes in us-CPR (n=8) (Figure 3 b ), the mRNA levels demonstrated an upregulation for *NLRP3* +1.0 FC (p=0.7384), and a downregulation for *NLRP1* and *IL-1*β of +1.0 FC (p=0.8587) and +1.0 FC (p=0.9795), respectively, when compared to individuals who did not present the change in us-CRP (n=12).

To demonstrate the power analysis of the studied transcripts according to the respective groups, the population size > 0.8 was considered sufficient. For the peripheral blood assay, the *NLRP1* gene had a power of 1. In the cell culture assay, *NLRP1* and *IL1B* genes had a power of 0.87 and 0.99, respectively. The other genes and groups not mentioned had G*power < 0.8.

## Discussion

The percentage of 88.2% of free trisomy cases in our study sample was slightly lower than 95.0% reported in the literature (Oster-Granite et al., 2011; Coppedè, 2016; Bull, 2020). This variance can be explained by differences in birth prevalence, which depend on maternal age, as well as the practice of prenatal diagnosis of DS followed by termination of affected pregnancies (Yaqoob et al., 2019).

Regarding the expression of *NLRP1, NLRP3* and *IL-1β* genes in individuals with DS, we found a downregulation of *NLRP1* when compared with the healthy control group. The *NLRP1* gene exhibited the most pronounced difference in expression in relation to the healthy control group and it was the only one that reached statistical significance. The downregulation of *NLRP1* and upregulation of *NLRP3* and *IL-1β* genes may indicate an unbalanced immune response in individuals with DS. Furthermore, upregulation of the *NLRP3* and *IL-1β* genes can influence upon chronic inflammation, potentially affecting the function of the endocrine system, possibly contributing to metabolic problems such as obesity, insulin resistance and diabetes.

According to Verstegen and Kusters (2020), given that all aspects of the immune system in individuals with DS present changes at some level, disrupted cellular communication may play a role in decreased function and dysregulation of gene expression. Consequently, the direct effect of this dysregulated expression can influence intracellular pathways, affecting fundamental cellular processes such as cell division and mitochondrial function, in addition to those that are specific to immune system cells.

Regarding the results of the mRNA levels of the *NLRP1*, *NLRP3* and *IL-1β* genes in individuals with hypothyroidism (n=9) compared to those without hypothyroidism (n=11), no statistically significant differences were observed. The most ordered tests in the diagnosis and treatment of hypothyroidism are thyroid peroxidase antibodies (anti-TPO) and thyroglobulin antibodies. Investigating these markers could help to identify autoimmune thyroiditis and provide a more comprehensive understanding of changes associated with the most common cause of hypothyroidism. Although autoimmune conditions such as type 1 diabetes mellitus (T1D), AITD, alopecia, celiac disease (CD), arthritis associated with Down syndrome, and vitiligo occur more frequently among individuals with DS compared to those without DS (Bull, 2020; Jones et al., 2020). In our sample, one individual (individual 8, Table 2) exhibited value above the reference range for (HbA1c value = 8.0% and EMG = 183 mg/dL), these elevated values are indicative of potential diabetes, particularly T1D, which is characterized by autoimmune destruction of insulin-producing beta cells in the pancreas. 

An increased expression of several inflammasome components, including *NLRP1, NLRP3, NLRC4, AIM2, ASC* and caspase-1, along with their downstream cytokines, namely *IL-18* and *IL-1β*, within thyroid tissues of patients with AITD was observed by Guo et al. (2018). This finding highlights the association of altered expression and activity of multiple inflammasomes with the pathogenesis of AITD in humans. A downregulation of the *NLRP1* and *NLRP3* genes in individuals with DM1 was also observed by Liu et al. (2017), which is an autoimmune condition often prevalent in individuals with DS. This finding underscores the complex and varied roles of inflammasomes in autoimmune disorders and their relevance in individuals with DS. Early recognition and treatment of autoimmune conditions can prevent complications throughout the lifespan. 

In our study, the analysis of serum levels of C-reactive protein (us-CRP), used as an important marker of inflammation because its levels are elevated in inflammatory conditions, including cardiovascular and autoimmune diseases (Yao et al., 2019), were detected in 8 individuals with DS. However, when we compared these individuals to those without changes in us-CRP levels, we did not find a statistically significant difference between the two groups. It is important to note, that a study conducted by Manti et al. (2018) investigated this marker in a group of individuals with DS compared to those without DS, they showed that ultrasensitive CRP (us-CRP) levels were significantly higher in individuals with DS. This underscores the significance of analyzing CRP levels in individuals with DS and highlights its potential relevance as an important marker in this context.

In conclusion, our analyses may suggest potential evidence between the altered expression of *NLRP1*, *NLRP3* and *IL1B* genes and susceptibility to immune-mediated diseases in individuals with DS. However, it is important to emphasize that more comprehensive analyses are needed to firmly establish the existence of this relationship. Additional research and investigations will be essential to confirm and elucidate the role of these genes in the context of autoimmune and inflammatory conditions in individuals with Down syndrome.
